# Advances in the diagnosis of myocarditis in idiopathic inflammatory myopathies: an overview of diagnostic tests

**DOI:** 10.1093/rheumatology/keae029

**Published:** 2024-01-16

**Authors:** Gautam Sen, Paul Scully, Patrick Gordon, Daniel Sado

**Affiliations:** Department of Cardiovascular Medicine, King’s College London, London, UK; School of Cardiovascular Medicine & Sciences, British Heart Foundation Centre of Excellence, King’s College London, London, UK; Department of Cardiology, King’s College Hospital NHS Foundation Trust, London, UK; Department of Nuclear Medicine, King’s College Hospital NHS Foundation Trust, London, UK; Department of Rheumatology, King’s College Hospital NHS Foundation Trust, London, UK; Department of Cardiovascular Medicine, King’s College London, London, UK; Department of Cardiology, King’s College Hospital NHS Foundation Trust, London, UK

**Keywords:** idiopathic inflammatory myopathies, cardiac biomarkers, creatine kinase, troponin I, cardiac magnetic resonance, ^18^F-FDG-PET/CT, endomyocardial biopsy

## Abstract

Cardiac involvement in idiopathic inflammatory myopathies (IIM) purports to worse clinical outcomes, and therefore early identification is important. Research has focused on blood biomarkers and basic investigations such as ECG and echocardiography, which have the advantage of wide availability and low cost but are limited in their sensitivity and specificity. Imaging the myocardium to directly look for inflammation and scarring has therefore been explored, with a number of new methods for doing this gaining wider research interest and clinical availability. Cardiovascular magnetic resonance (CMR) with contemporary multiparametric mapping techniques and late gadolinium enhancement imaging, is an extremely valuable and increasingly used non-invasive imaging modality for the diagnosis of myocarditis. The recently updated CMR-based Lake Louise Criteria for the diagnosis of myocarditis incorporate the newer T1 and T2 mapping techniques, which have greatly improved the diagnostic accuracy for IIM myocarditis.^18^F-FDG-PET/CT is a well-utilized imaging modality in the diagnosis of malignancies in IIM, and it also has a role for the diagnosis of myocarditis in multiple systemic inflammatory diseases. Endomyocardial biopsy, however, remains the gold standard technique for the diagnosis of myocarditis and is necessary for the diagnosis of specific cases of myocarditis. This article provides an overview of the important tests and imaging modalities that clinicians should consider when faced with an IIM patient with potential myocarditis.

## Graphical abstract

**Figure:**
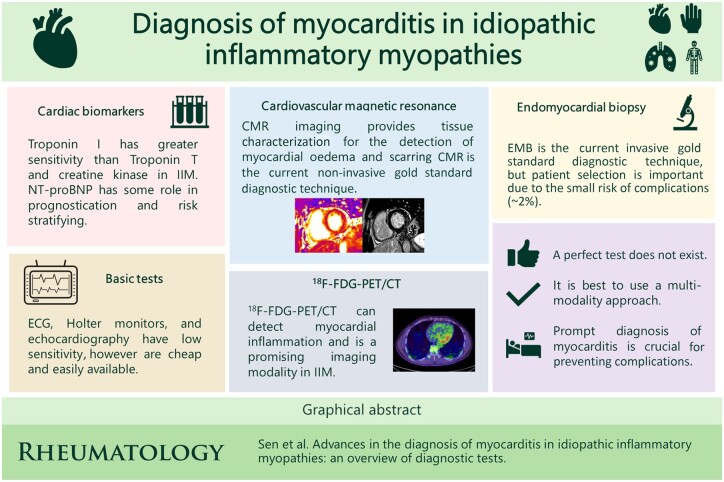

Rheumatology key messages

- Early recognition of myocarditis in idiopathic inflammatory myopathies can help initiate targeted treatment to prevent worse clinical outcomes.
- Cardiovascular magnetic resonance and endomyocardial biopsy are important tools in the detection of myocarditis and are the current non-invasive and invasive gold standard techniques, respectively.
- There is no one perfect test for detecting myocarditis; therefore, a strategy combining cardiac biomarkers (troponin I) with non-invasive imaging modalities (especially CMR) is essential for getting an earlier diagnosis.
- The future in this field is exciting, and there are other imaging modalities, such as ^18^F-FDG-PET/CT, that have shown great promise in detecting myocardial inflammation in other autoimmune diseases.

## Introduction

Idiopathic inflammatory myopathies (IIMs) are rare systemic inflammatory diseases [1, 2] and include DM, PM, IBM, anti-synthetase syndrome (ASA) and immune-mediated necrotizing myopathy (IMNM) [2, 3]. Proximal muscle weakness and myalgia are the commonest presenting symptoms; however, they are multisystem disorders, sometimes involving cutaneous, cardiovascular, pulmonary, and gastrointestinal systems [2, 4, 5]. The three leading causes of death are malignancy, interstitial lung disease (ILD) and cardiac disease, with the accumulation of extramuscular manifestations correlating with worse clinical outcomes [4, 6].

### Cardiac involvement in IIM

IIMs are rare, and data on cardiac involvement is limited. Cardiac disease has been the leading cause of death in several studies [7–9]. A study of 162 patients, with a median follow-up time of 101.5 months, showed that cardiac involvement was the main prognostic factor for death [10]. Cardiac involvement can be due to myocardial inflammation, which can then cause myocardial scarring [2, 11]. This can cause heart failure and conduction disease, which can result in malignant arrhythmia and death [11–13]. In addition it is now recognized that accelerated atherosclerotic coronary artery disease is common [14]. Recent work at our centre found an increased risk of cardiovascular events in the first 5 years after diagnosis [15]. This risk is comparable with the increase in risk seen in RA patients [16]. Typical cardiac manifestations in IIM are summarized in Supplementary Table S1, available at *Rheumatology* online. The rest of this review will focus on the detection of myocarditis in IIM.

### Myocarditis in IIM

Myocarditis is defined as an inflammatory injury to the myocardium resulting from a number of potential causes ranging from infection, autoimmune disease, drugs, and inherited diseases [17, 18]. The differential diagnosis of causes is important to consider in the work-up of myocarditis, with the most common aetiology being viral infection [19].

Myocarditis in the IIMs is frequently subclinical [20]; however, this does not mean it is benign. Uncontrolled myocardial inflammation can potentially progress to myocardial fibrosis, leading to heart failure, arrhythmia, and worse clinical outcomes [13]. A study of ASA patients with myocarditis showed that 50% needed intensive care treatment [21]. A more recent paper showed that myocarditis in IIM patients leads to poor outcomes, despite immunosuppression, possibly due to late detection [22]. As such, a proactive approach to accurately diagnosing myocarditis is important to guide immunosuppressant therapy. However, its recognition is often delayed in clinical practice, partly because the disease process is slow, insidious, and initially asymptomatic [2]. Perhaps more importantly, conventional investigations such as ECG and echocardiography have low sensitivity and specificity. In a meta-analysis, the incidence of cardiac involvement in patients with PM/DM ranged between 9% and 72% [20]. This reflects the variation in screening methods used and inclusion of minor rhythm abnormalities of dubious significance (sinus tachycardia), which is a non-sensitive/non-specific finding.

With the limitations of current investigations, efforts have focused on finding better ways to assess for myocarditis. Testing for blood biomarkers have the advantage of wider availability and limited expertise required to measure them. However, they are often limited in specificity. Non-invasive imaging modalities to directly look for myocardial inflammation and scarring have, therefore, been explored, with a number of new methods for doing this gaining wider research interest and increased use. These methods include cardiovascular magnetic resonance (CMR) and ^18^F-fluorodeoxyglucose (FDG)-PET/CT (^18^F-FDG-PET/CT) and will be discussed in the following sections. The gold standard approach, however, remains myocardial histology by endomyocardial biopsy (EMB).

## Diagnosis of myocarditis in IIM

In this section, we review the important tests to consider, highlighting their advantages and limitations, starting with blood biomarkers, ECG and Holter monitoring, non-invasive imaging tests such as echocardiography, CMR and ^18^F-FDG-PET/CT, and finally EMB. Supplementary Table S2, available at *Rheumatology* online, summarizes these diagnostic tests.

## Clinical symptoms

The clinical presentation of myocarditis in IIM can vary, and some individuals may be asymptomatic [20]. ‘Red flag‘ symptoms include chest pain, fatigue, dyspnoea, palpitations and fever [23]. Myocarditis can lead to heart failure, which may present with serious complications such as pulmonary oedema, tachyarrhythmias, or sudden death [24]. The risk of sudden death is generally higher in cases of severe myocarditis, and factors such as the underlying cause and the extent of inflammation can influence patient outcomes.

## Serum biomarkers

### Creatine kinase

Creatine kinase (CK) has been used for decades to assess skeletal muscle inflammation [2, 25]. CK is also present in cardiac muscle and, before the advent of troponin (Tn), was used as a key biomarker in the diagnosis of acute coronary syndromes (ACSs) and myocarditis. However, it has poor sensitivity compared with Tn and is rarely used for this indication in contemporary clinical practice.

### Troponin T and troponin I

The use of Tn as a biomarker for myocardial injury is well established [26]. It has a key role in the diagnosis of ACS and myocarditis; however, it has limited specificity [27]. Cardiac conditions such as aortic dissection and cardiac trauma, and non-cardiac conditions such as pulmonary embolism, sepsis, and renal failure can lead to elevation in Tn [28, 29].

Tn T (TnT) levels are often raised in patients with active IIM, as TnT is expressed in regenerating skeletal muscle cells [30, 31]. As such, TnT has limited specificity as a biomarker for ACS and myocarditis in patients with active muscle disease. In fact, the use of TnT can lead to inappropriate cardiac investigations [32–34]. In contrast, however, Tn I (TnI) is not expressed in regenerating skeletal muscle cells and is more specific to the myocardium [35]. Due to the better specificity of TnI over TnT for myocardial damage, it is likely to be a better marker for the identification of myocarditis [36].

One study noted that TnI levels did not differ in IIM patients compared with healthy controls [37]; however, other studies have reported that patients with elevated TnI levels had abnormalities on non-invasive cardiac investigations, including ECG and echocardiography [38, 39]. One study assessed TnI levels in patients with CMR-confirmed myocarditis and found it to have high sensitivity (97%) and specificity (84%) for cardiac involvement [40]. The use of TnI as a biomarker to assess myocardial involvement is still novel and requires further investigation.

### N-terminal pro-brain natriuretic peptide

N-terminal pro-brain natriuretic peptide (NT-proBNP) is often elevated in myocarditis, and is released in response to increased stretching of the heart muscle [41]. Higher baseline NT-proBNP levels were an independent predictor of poor outcomes in patients with acute myocarditis and may, therefore, be a useful biomarker for risk stratification [42].

## Basic investigations: ECG and Holter monitoring

### ECG

The 12-lead ECG is widely available and cheap and is performed on all patients with the potential for a cardiac diagnosis. ECG abnormalities can be seen in up to 75% of IIM patients [20, 43]. The most common abnormalities are ST-T abnormalities, bundle branch blocks and ventricular premature complexes; however, prolongation of the QTc interval has been noted [37, 44, 45]. Many of these findings are non-specific, and this, along with limited sensitivity for subclinical myocarditis, means that ECGs are not a very useful test for the diagnois of myocarditis in IIM.

### Holter monitoring

Prolonged ECG monitoring (often over a 24-h period) is the key method used to detect paroxysmal arrhythmia. Since, myocarditis may present, with the risk of sudden death due to arrhythmias, this is an important test in the diagnostic process to help with risk stratification [46]. Holter monitoring has a role in prognostication in myocarditis patients. Some ECG abnormalities that are independent predictors of adverse prognosis, such as broad QRS complexes and prolonged QT intervals, may play an important role in deciding appropriate therapy and the timing of further intervention, such as implantable defibrillators [47]. Only one study has evaluated this using 48-h Holter monitoring, with the only abnormality being higher heart rates in IIM patients compared with healthy controls [38].

## Non-invasive imaging modalities: echocardiography, CMR and ^18^F-FDG-PET/CT

### Echocardiography

Echocardiography is widely available and is a relatively cheap method for detecting functional and structural abnormalities. Echocardiographic abnormalities are reported in up to 60% of patients with IIM [48–50]. However, these findings are of limited sensitivity and specificity, because ultrasound is limited in myocardial tissue characterization. For example, ventricular dysfunction on echocardiography is not specific for myocarditis; nor does it differentiate active disease from scarring from prior damage. Equally, echocardiography may be normal in the presence of significant myocarditis if it has not impacted ventricular function [51, 52].

While less routinely used in clinical practice, the addition of speckle-tracking echocardiography (STE) and global longitudinal strain (GLS) may be of more use than routine echocardiography. STE is a non-invasive imaging technique used to assess myocardial strain and deformation [53]. It helps assess myocardial function and detect regional abnormalities. GLS is a newly emerging echocardiographic tool that has a significant role in predicting cardiovascular outcomes beyond that of measuring just ejection fraction [54]. When investigated using STE, IIM patients with preserved left ventricular ejection fraction had reduced biventricular strain and lower GLS when compared with healthy controls matched for age and sex [55–57]. Reduced strain and lower GLS are both markers of reduced myocardial contractility and can give further information of early cardiac involvement in patients with a normal ejection fraction.

### Cardiovascular magnetic resonance

CMR facilitates non-invasive characterization of myocardial tissue and is strongly recommended as a Class I assessment tool for myocarditis [58, 59]. Since 2009, the diagnosis of myocarditis by CMR has relied on the Lake Louise criteria (LLC), addressing myocardial inflammation through considerations of oedema, hyperaemia, necrosis, and/or fibrosis [60]. The initial LLC interpretation involved a qualitative or semiquantitative analysis of signal intensities on T2-weighted, early gadolinium enhancement, and late gadolinium enhancement (LGE) images. However, the original LLC’s diagnostic accuracy was constrained when significant signal intensity distinctions between affected and normal myocardium were lacking, potentially leading to oversight in cases of diffuse or subtle myocardial inflammation.

T1 and T2 mapping are robust CMR techniques and provide a pixel-wise quantitative characterization of myocardial tissue [60, 61]. T1 and T2 mapping techniques have been shown in numerous studies to have clear benefits when compared with the original LLC in evaluating inflammation [62, 63]. The LLC criteria were, therefore, modified in 2018 to include mapping techniques. The 2018 LLC criteria say that CMR-based diagnosis of myocarditis now requires the presence of at least one T1-based criterion (increased myocardial T1 relaxation times, extracellular volume fraction, or LGE) in combination with at least one T2-based criterion (such as increased myocardial T2 relaxation times, myocardial oedema, or an elevated T2 signal intensity ratio) [60, 63].

In cases of acute myocarditis, the diagnostic accuracy of the LLC reaches ∼80%. However, there is a potential for underestimating the prevalence of subtle myocarditis, posing a risk of false-negative results, especially in patients with concurrent myositis, a notable concern within the IIM population [64]. To address these challenges, the use of the CMR mapping approach emerges as a promising solution. This approach offers quantitative maps of myocardial relaxation times, providing insights into structural and pathological alterations within the myocardium. Consequently, this advanced technique aids in distinguishing areas of active inflammation from those in the healing phase [65].

This section explains these techniques in more detail and how they are being utilized for the diagnosis of myocarditis in IIM.

### Late gadolinium enhancement

An important factor in the LLC criteria is LGE, used to identify focal myocardial scarring. It has been used to detect myocardial infarction, and viability in areas of abnormal wall motion, and for the assessment of scarring caused by non-ischaemic diseases such as myocarditis [60, 66]. Gadolinium is an extracellular tracer—it is small enough to diffuse out of capillaries, but too large to enter into cells with an intact cell membrane. Therefore, following i.v. administration, more gadolinium contrast will collect in areas of extracellular space expansion than in the surrounding normal myocardium. After a few minutes post i.v. administration, areas of myocardial scarring will contain more gadolinium than the surrounding normal myocardium, resulting in lower T1 signal in the areas of scarring. MRI sequences that are T1 weighted are then used to detect this change, resulting in an image in which the areas of scarring are white (and hence said to ‘late enhance‘), and the normal areas of myocardium surrounding these areas are black.

**Figure 1.**
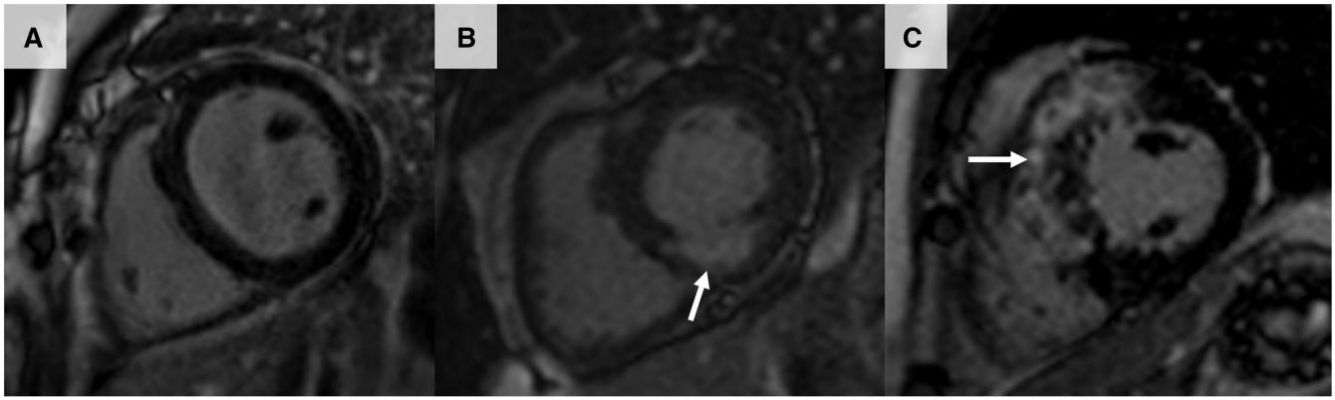
Cardiac magnetic resonance late gadolinium enhancement patterns showing ischaemic and non-ischaemic pathologies

**Figure 2.**
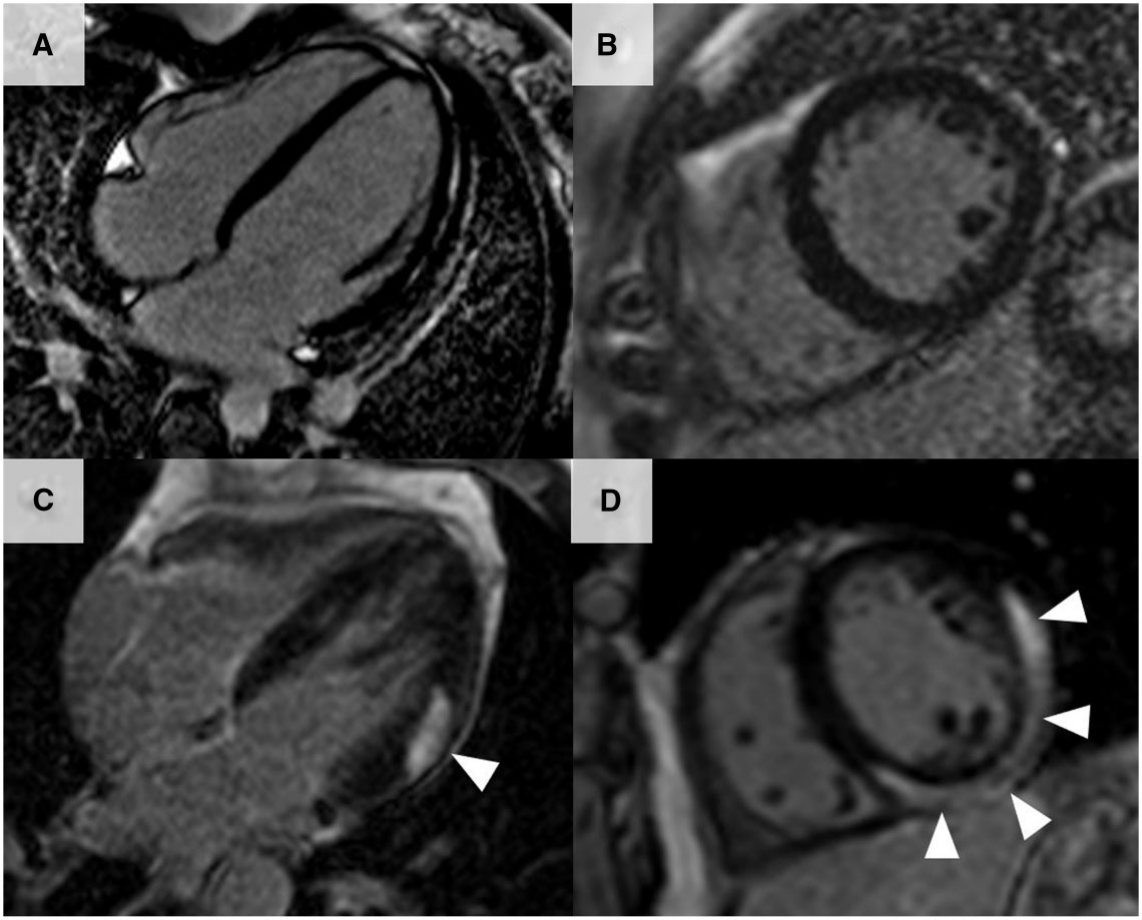
Cardiac magnetic resonance late gadolinium enhancement pattern showing normal myocardium compared with myocarditis

The location, distribution, and transmural extent of LGE within the myocardium allows differentiation between the various causes of cardiac pathology, such as between ischaemic and non-ischaemic disease (Fig. 1). The presence and burden of LGE is prognostically important in non-ischaemic cardiomyopathies [66, 67], including dilated cardiomyopathy [68], hypertrophic cardiomyopathy [69], and cardiac amyloidosis [70], and can act as a nidus for malignant arrhythmias and be a harbinger of sudden cardiac death. The LGE pattern associated with myocarditis is predominantly subepicardial/mid-wall in nature and is localized most frequently in the lateral or inferior walls (Fig. 2) [11, 71].

### Limitations

While LGE has proven to be a valuable tool, it does have limitations. It relies on the delayed wash-out of gadolinium-based contrast agents from normal myocardium, leading to greater contrast between scarred and healthy tissue [72]. The optimal timing for image acquisition is critical. If the imaging is performed too early or too late after contrast administration, the contrast between scarred and normal tissue may be diminished, leading to reduced diagnostic accuracy [73]. The detection of small or subtle areas of scar can be challenging due to limitations in spatial resolution. Finally, LGE requires the administration of gadolinium-based contrast agents, which carries a small risk of adverse reactions [74]. In patients with renal insufficiency (estimated glomerular filtration rate < 30 ml/min), there may be a small increase in risk of nephrogenic systemic fibrosis.

### CMR parametric mapping

CMR mapping was developed to overcome some of the limitations of conventional MRI sequences and provide more detailed and quantitative information about myocardial tissue. Mapping offers several advantages over LGE in certain clinical scenarios by providing direct measurements of tissue properties, such as T1 and T2 relaxation times. This allows for a more objective and precise assessment of tissue characteristics compared with the semi-quantitative information provided by LGE [61, 75]. Quantitative measurements can be helpful in tracking changes over time, monitoring treatment response, and comparing tissue properties within and between patients. Mapping techniques for the assessment of myocarditis are T1, T2 and extracellular volume (ECV) mapping [61].

### T1 mapping

T1 mapping sequences allow quantitative evaluation of T1 in each of the 12 basal to mid left ventricular myocardial segments (it is less reliable in the assessment of the apical segments). The values obtained can then be compared with normal ranges. T1 will increase in areas of oedema, scarring and infiltration. For this reason, it is useful for detecting myocardial inflammation in acute states, but also for chronic pathologies in which the myocardium has an expanded interstitial space where free water can accumulate, such as in areas of chronic fibrosis (scarring) [61, 76].

**Figure 3.**
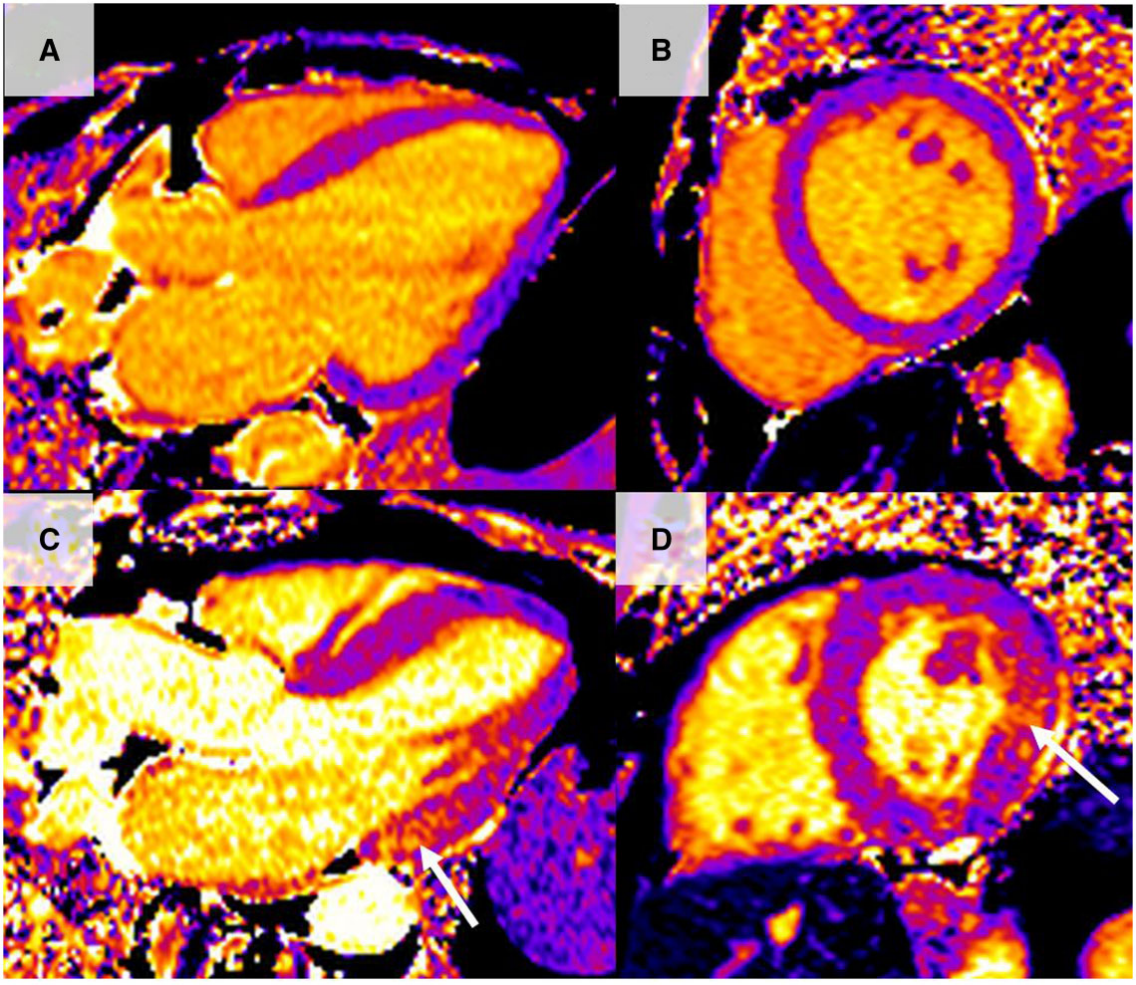
Cardiac magnetic resonance T1 mapping. (A and B) Long axis 3-chamber and short axis views showing normal T1 values within the myocardium. (C and D) Long axis 3-chamber and short axis views showing elevated T1 values at the basal lateral wall (peak T1 value of 1300 ms, with normal <1100 ms) (white arrows) in a patient with SSc, consequently diagnosed with myocarditis. The high signal at the lateral wall within the myocardium shows high T1 signal compared with the rest of the myocardium

As such, T1 mapping has shown clinical utility for detecting changes in many conditions, including acute myocardial infarction, acute myocarditis, and cardiac amyloidosis, as these have high native T1 values [61, 77]. As a result, T1 mapping is increasingly being used to detect cardiac involvement in rheumatological diseases (Fig. 3). T1 mapping has advantages in being able characterize myocardial tissue without needing to administer contrast. It also allows for the assessment of diffuse and early myocardial disease, which is much more difficult to assess using conventional MRI techniques. The quantitative nature of mapping also offers the potential to allow for monitoring of changes with therapy, both in clinical and academic domains.

### T2 mapping

**Figure 4.**
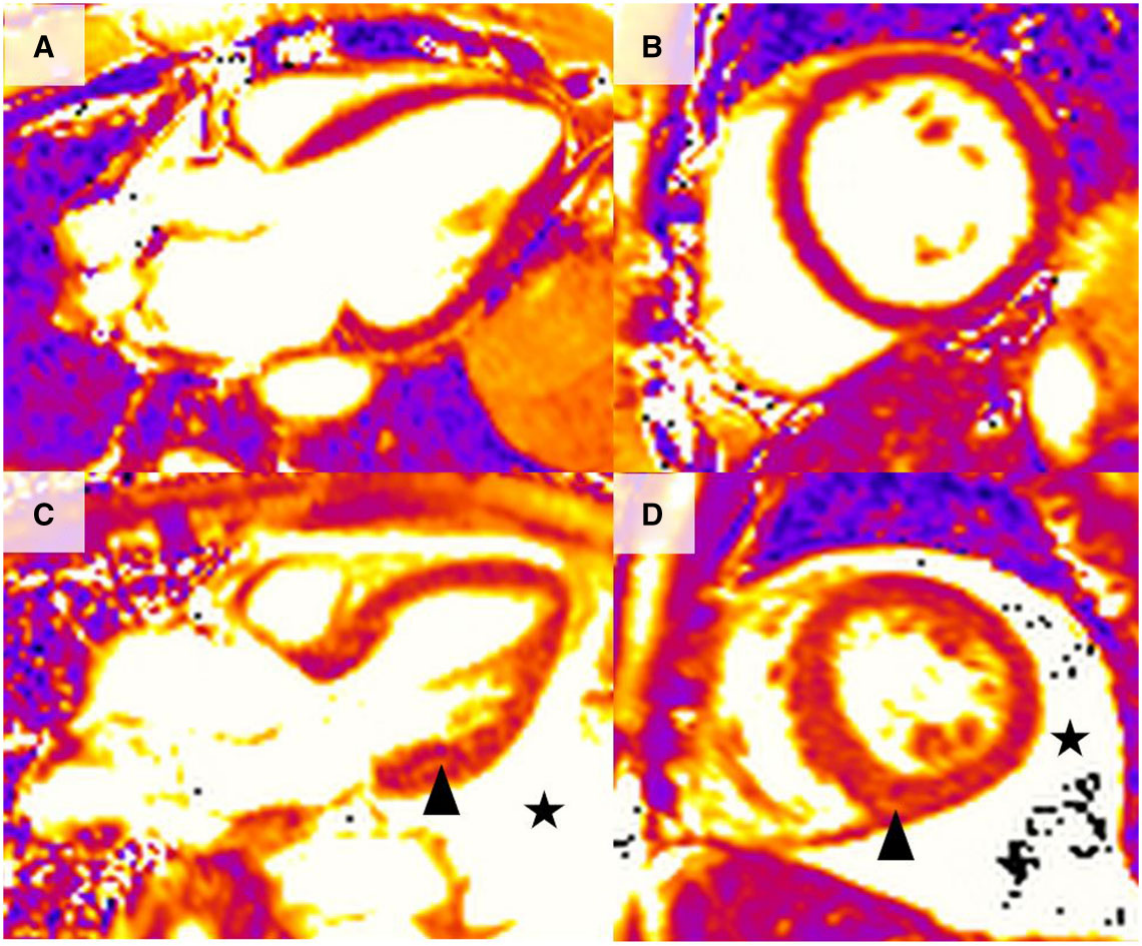
Cardiac magnetic resonance T2 mapping. (A and B) Long axis 3-chamber and short axis views showing normal T2 values within the myocardium. (C and D) Long axis 3-chamber and short axis view of a patient with SLE consequently diagnosed with myocarditis. There is diffuse T2 elevation in the whole myocardium, with a peak seen at the basal inferior/infero-lateral walls (black arrowheads), with an elevated T2 value of 65 ms (normal value <48ms). Elevation of T2 is a marker of acute myocardial inflammation and oedema, often seen in acute myocarditis. There is also a large pericardial effusion (black star)

T2 mapping sequences allow quantitative evaluation of T2 in each of the 12 basal to mid left ventricular myocardial segments (as with T1 mapping, it is also less reliable in the assessment of the apex). T2 is much more specific for the assessment of acute oedema and therefore has been utilized in the study of acute myocardial infarction, myocarditis, and sarcoidosis [78–80]. Regional and global T2 times are significantly increased in patients with myocarditis, and longer T2 relaxation times correlate with myocardial oedema and inflammation seen in endomyocardial biopsy samples [81]. This is a particularly helpful utility of T2 mapping, as it can help discriminate between active and healed myocarditis, with T2 values normalizing in healed myocarditis [65]. Furthermore T2 mapping has been utilized in rheumatological diseases such as SSc, greatly improving the sensitivity for detecting myocardial inflammation [64]. As with T1 mapping, the quantitative nature of the technique allows for the assessment of both focal and diffuse myocardial disease processes and has potential for monitoring of therapy. T2 mapping is therefore increasingly used to detect cardiac involvement and myocardial oedema in rheumatological diseases (Fig. 4).

### ECV mapping

ECV is a technique used to assess myocardial tissue composition and fibrosis. ECV measurement uses T1 mapping prior and post gadolinium contrast administration [82]. ECV is a quantitative measurement represented as a percentage. It represents the fraction of the myocardium that is composed of extracellular space, which can increase due to fibrosis or other pathological changes. CMR with ECV is used in the evaluation of various conditions, including myocardial infarction [83], cardiomyopathies such as dilated cardiomyopathy [84], myocarditis [85], and SSc [64, 86].

### CMR studies in IIM

Novel CMR techniques have shown clinical utility in the detection of myocardial inflammation and fibrosis in several autoimmune conditions, including RA [48, 75, 87], SSc [64, 86, 88, 89], SLE [90, 91] and sarcoidosis [92]. These sequences have also been used with IIM patients, often demonstrating CMR abnormalities.

A few studies found a reduction in left ventricular systolic function; however, the majority showed preserved systolic function [37, 93]. A common finding from the studies was the presence of LGE showing myocardial scarring. Four studies evaluating LGE in IIM patients without previously diagnosed myocarditis demonstrated it in 20–62% of patients, compared with age- and sex-matched healthy controls [37, 38, 93–96]. The location of LGE was mostly non-ischaemic, suggesting myocarditis as the most likely aetiology.

**Figure 5.**
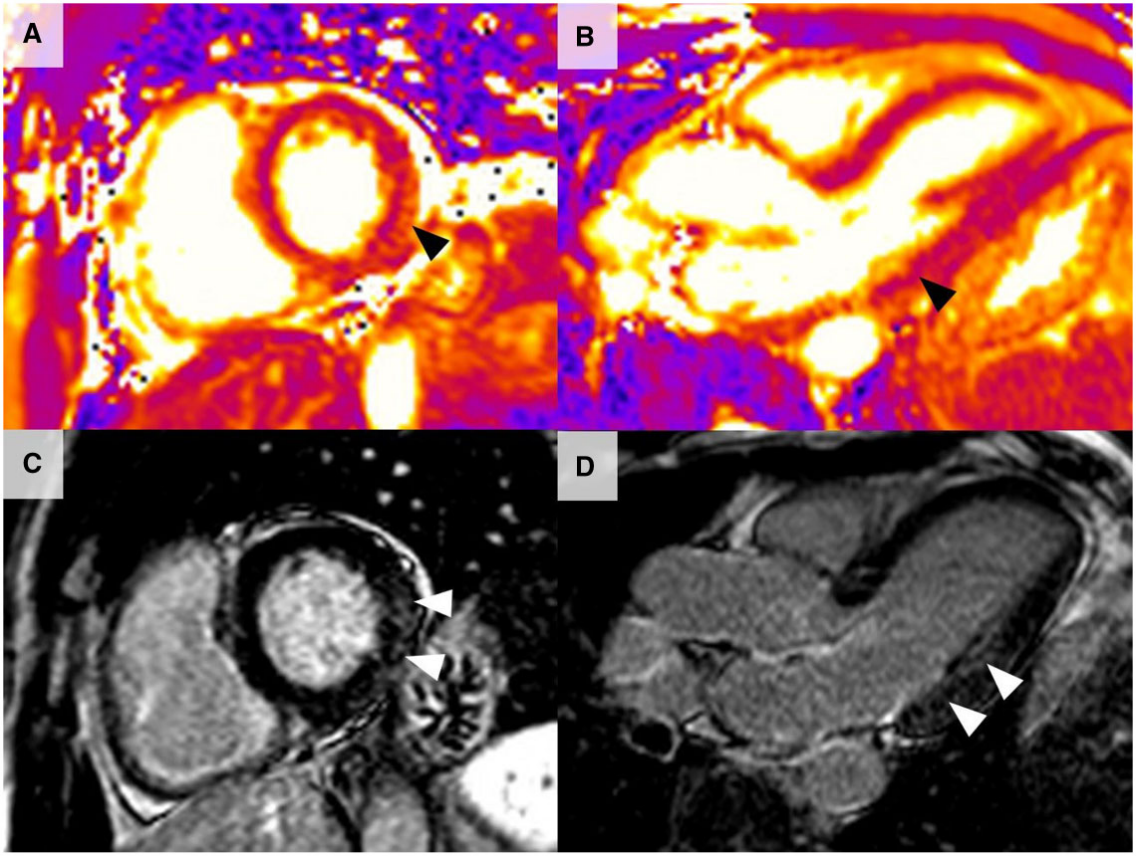
Cardiac magnetic resonance in an IIM patient. (A and B) Short axis and long axis 3-chamber T2 mapping images showing elevated myocardial T2 values in the basal lateral wall (black arrows). (C and D) Short axis and long axis 3-chamber late gadolinium images showing mid wall late enhancement in the basal lateral wall (white arrows). Elevation of T2 is a marker of acute myocardial inflammation, often seen in acute myocarditis. Late enhancement in the mid wall is a classic finding in myocarditis

Studies that have used T1 mapping have in general showed an elevation of T1 mapping values and ECV abnormalities, suggesting increased myocardial fibrosis and/or oedema compared with healthy controls [37, 38, 93–97]. Elevation of T2 mapping values was also seen in several studies, compared with healthy controls, suggesting myocardial oedema and inflammation [88, 92–94]. T2 values were elevated even in patients with normal left ventricular ejection fraction and in the absence of LGE [95, 98]. Myocardial T2 values were elevated in both IIM patients and myocarditis patients, and could distinguish patients from healthy controls. However, T2 mapping could not on its own differentiate the cause of myocarditis, and could not distinguish between an autoimmune aetiology and a viral cause [98]. In addition, IIM patients undergoing treatment were noted to have lower T2 values that paralleled improvement in serum inflammatory markers, suggesting that T2 mapping might have utility as a monitoring tool [98, 99] (Fig. 5 shows a case of IIM myocarditis from our centre).

Many of these CMR abnormalities have been demonstrated in asymptomatic patients, suggesting subclinical myocardial involvement. The longer-term implications of these abnormal findings are unclear at present; however, it is well established that the presence of LGE and abnormal mapping parameters are associated with poorer outcomes [23, 67–70, 81]. For this reason, it is paramount that longitudinal studies are performed to confirm whether the presence of LGE or elevated mapping parameters predicts a poorer prognosis in the IIM cohort.

### Advantages and limitations of CMR

One of the advantages of CMR over other modalities is safety. It is non-invasive, has no radiation exposure, and can be used for repeated scans of the same patient. A study using CMR in IIM patients showed that it can be used safely and effectively as a monitoring tool, with cardiac abnormalities reducing after treatment with CSs [100]. This is an exciting potential use of CMR in the IIM population, as it would enable patients to undergo repeated scans to assess response to treatment [79, 101, 102].

There are a few limitations of CMR that need to be considered. CMR is an expensive imaging modality, and for this reason it is not widely available in some countries. It requires great expertise in performing and interpreting optimal scans. Furthermore, at present there is no prognostic data for myocarditis in IIM patients.

## PET

PET is a nuclear medicine imaging technique that uses a radioactive tracer, typically a form of glucose labelled with a positron-emitting isotope like fluorine-18 fludeoxyglucose (^18^F-FDG) [103]. Due to inflammation, immune cells and tissues become more metabolically active, leading to an increased uptake of glucose. By injecting ^18^F-FDG into the patient’s bloodstream, areas of heightened metabolic activity are highlighted, indicating regions of inflammation. For this reason, ^18^F-FDG-PET/CT is a well-utilized imaging modality in the diagnosis of malignancies [104].

**Figure 6.**
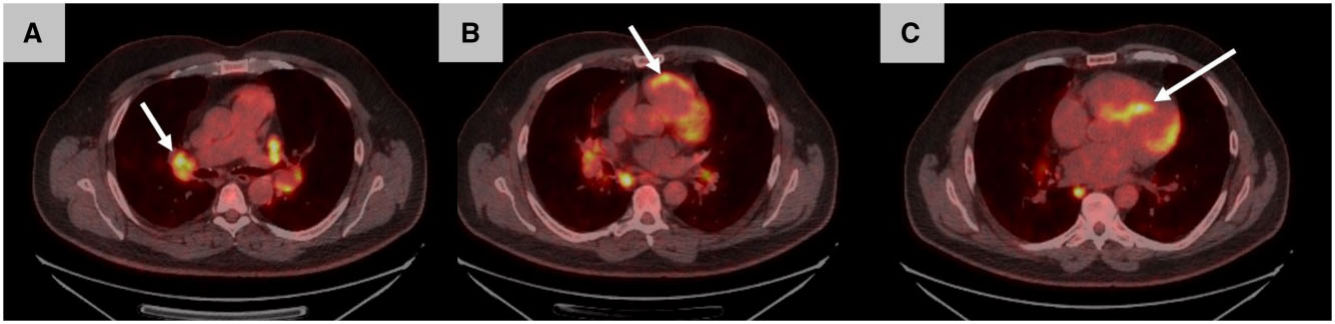
18-FDG-PET/CT showing cardiac sarcoid. 18-FDG-PET/CT transverse images showing multiple intensely avid areas of FDG uptake (A) in mediastinal and hilar lymph nodes, (B) in the right ventricle and (C) in the left ventricle, with the anteroseptum showing the highest level of FDG uptake. A diagnosis of cardiac sarcoidosis was made. 18-FDG-PET/CT: fluorine-18 fluorodeoxyglucose PET/CT; FDG: fluorodeoxyglucose.

^18^F-FDG-PET/CT is an important diagnostic test in cardiac inflammatory diseases such as sarcoidosis [105, 106]. CMR and ^18^F-FDG-PET/CT evaluate different pathological processes: CMR primarily evaluates fibrosis using LGE, and ^18^F-FDG-PET/CT primarily evaluates inflammation using labelled-glucose uptake of activated macrophages (Fig. 6). Neither technique is specific for fibrosis or inflammation caused by cardiac sarcoidosis. As such, these different imaging modalities can provide complementary information [107].

^18^F-FDG-PET/CT is not yet used routinely for the detection of cardiac disease in rheumatological diseases such as RA and SSc; however, a few studies have shown it to be promising [108, 109]. In these conditions, it has detected subclinical cardiac disease and has demonstrated improvements after therapy. At present ^18^F-FDG-PET/CT only has a limited role in the diagnosis and monitoring of myocarditis; however, recent studies have shown its clinical utility, especially when CMR is unsuitable because of an irregular heartbeat or artefacts caused by implantable devices [110, 111].

In the IIMs, ^18^F-FDG-PET/CT is used to identify malignancies (which can particularly be found in DM patients) [112]. An advantage of ^18^F-FDG-PET/CT imaging is that it provides whole-body imaging and can evaluate metabolically active extracardiac features, such the status of skeletal myopathy, as well as detecting ILD. ^18^F-FDG-PET/CT is also favoured over CMR for patients with advanced kidney disease, as administration of gadolinium is contraindicated in these patients.

A disadvantage of using ^18^F-FDG-PET/CT is that it requires adequate preparation by adhering to a specific high-fat, low-carbohydrate diet and fasting for 18 hours before the scan [113]. This preparation is designed to suppress normal myocardial glucose uptake, enabling more accurate detection of inflammatory activity within the myocardium [107]. However, it is predicted that 10–15% of cardiac studies fail due to poor suppression of physiologic glucose uptake [114]. Furthermore, ^18^F-FDG-PET/CT involves the use of ionizing radiation, which should be considered, particularly when performing interval imaging.

There are currently no studies of the use of ^18^F-FDG-PET/CT to diagnose myocarditis in IIM, and further research is needed.

## Endomyocardial biopsy

EMB remains the gold standard for diagnosing myocarditis, in accordance with various guidelines [115, 116]. By directly examining myocardial tissue, it allows identification of inflammatory changes and the presence of infiltrating immune cells, providing concrete evidence of myocardial inflammation.

EMB has a crucial role in the diagnosis of myocarditis when the underlying aetiology is unclear [117]. Individuals with IIM, like many with rheumatological diseases, often receive steroids or immunosuppressants, necessitating careful consideration of the heightened risk of infectious or viral myocarditis in immunocompromised patients [118]. In this context, EMB plays a pivotal role in excluding a viral aetiology, through RT-PCR analysis of heart specimens, crucial for ruling out the presence of cardiotropic viral genomes [117]. This has several diagnostic and therapeutic implications and is necessary for diagnosing certain forms of myocarditis that require targeted therapies, such as in sarcoidosis, eosinophilic and giant cell myocarditis.

Complications can occur rarely, and the decision to perform EMB should be carefully considered based on the patient’s clinical condition, the expertise of the medical team, and potential benefits *vs* risks. The risks can include cardiac tamponade, arrhythmias, heart block, pneumothorax, and pulmonary embolization [18, 119]. In centres where there is a high level of experience in EMB, it can be performed with major complication rates of <2% [117].

In spite of the low risk of complications, EMB is rarely performed in IIM, in our experience. There are only a few studies that have published data on EMB-confirmed myocarditis in patients with IIM, within cohorts of individuals with various CTDs [64, 120]. This rarity could be attributed to its low sensitivity, stemming from potential sampling errors in what is often a heterogeneous involvement of the myocardium. However, recent studies have clearly demonstrated that right-heart EMB, when conducted in expert centres, can exhibit substantial diagnostic accuracy in confirming left-sided myocarditis as evidenced by CMR [121].

## Diagnostic algorithm for detecting myocarditis in IIM

IIM myocarditis can be a potentially serious complication. After undertaking focused history for cardiac symptoms, investigations are warranted if there are any underlying concerns for myocarditis. Basic investigations include ECG, Holter monitoring, and blood tests, including TnI. Non-invasive cardiac imaging should be performed, which will be guided by cost and availability. An echocardiogram should be the first test to look for left ventricular dysfunction, due to its low cost and availability, followed by CMR, as the current gold standard for non-invasive tissue characterization. The CMR imaging protocol should include cine imaging to determine left ventricular volumes and function, followed ideally by parametric mapping techniques to detect myocardial fibrosis and oedema, and lastly LGE imaging to detect scarring. ^18^F-FDG-PET/CT is a useful test, as it can potentially give information about cardiac involvement, as well as extra-cardiac manifestations of IIM, such as ILD, malignancy, and the degree of skeletal involvement. If the diagnosis of myocarditis remains inconclusive, or a more potent form of myocarditis is suspected, EMB should be considered in order to provide a definitive diagnosis. There are currently no published guidelines for the diagnosis of IIM myocarditis; however, it is important that a combination of these available diagnostic modalities are utilized to make an accurate and prompt diagnosis.

## Conclusion

Cardiac involvement is well recognized in the IIMs and leads to worse clinical outcomes. Detecting cardiac involvement, especially myocarditis, early in the disease process can help initiate targeted treatment. This is essential to prevent the development of cardiac complications such as heart failure and arrhythmias. There is no one perfect test to detect myocarditis in IIM; therefore, a strategy combining testing for cardiac biomarkers with non-invasive imaging modalities, especially CMR, is essential to getting an earlier diagnosis. EMB should be considered in special cases and remains the gold standard. The future in this field is, however, exciting, and there are other imaging modalities such as ^18^F-FDG-PET/CT that have shown promise in detecting myocardial inflammation in other autoimmune diseases.

## Supplementary Material

keae029_Supplementary_Data

## Data Availability

No new data were generated or analysed in support of this article.
