# Supplementary material for: Advances in the diagnosis of myocarditis in idiopathic inflammatory myopathies: an overview of diagnostic tests
Source: Rheumatology (Oxford). 2024 Jan 16;63(7):1825–36. doi: 10.1093/rheumatology/keae029 (PMC11215992; doi:10.1093/rheumatology/keae029)
Supplement: keae029_Supplementary_Data [file keae029_supplementary_data.docx]

**Supplementary Table S1: Cardiac manifestations seen in the idiopathic inflammatory myopathies**

| **Cardiac manifestations seen in the idiopathic inflammatory myopathies** |
| --- |
| Heart failure with preserved or reduced left ventricular systolic function  Accelerated atherosclerosis and myocardial infarction  Left atrial enlargement  Left ventricular hypertrophy  Myocarditis  Conduction disorders (including LBBB and heart blocks)  Arrhythmias (including supraventricular tachycardias such as atrial fibrillation)  Sudden cardiac death |

**Supplementary Table S2: Advantages and limitations of different tests and imaging modalities to diagnosis myocarditis in idiopathic inflammatory myopathy.**

|  | **Test or imaging modality** | **Advantages** | **Limitations** |
| --- | --- | --- | --- |
| **Cardiac biomarkers** | **Creatine kinase** | - Easily available - Useful to detect skeletal involvement | - Not specific for cardiac disease. Elevated levels can be due to skeletal muscle involvement. |
|  | **Troponin T** | - Easily available | - Not specific for cardiac disease. Elevated levels can be due to skeletal muscle involvement. |
|  | **Troponin I** | - More specific for cardiac involvement than other biomarkers |  |
|  | **NT-proBNP** | - Has a potential role in prognostication | - Not specific for myocarditis |
| **Basic investigations** | **ECG (12-lead)** | - Cheap - Easily available | - Low sensitivity and specificity |
|  | **Holter monitoring** | - Cheap - Has some role in prognostication | - Low sensitivity and specificity |
| **Non-invasive imaging modalities** | **Echocardiography** | - Easily available - Useful as a primary test to assess LV function - Some advanced techniques now available which might give further information regarding contractility | - Low sensitivity and specificity - Does not perform tissue characterisation - In certain patients can give poor quality images |
|  | **CMR** | - Current non-invasive diagnostic modality for myocarditis - Can perform tissue characterisation (T1 mapping, T2 mapping, LGE) which detects myocardial inflammation and scar - No radiation - Overall high-resolution images - Easy to perform as a monitoring tool due to safety and high level of reproducibility | - Expensive - Not available at all centres - Requires expertise to perform and interpret - Very small risk of contrast reaction |
|  | **PET/CT** | - Excellent at detecting myocardial inflammation (however limited data in IIM cohort) - Overall high-resolution images - Can give extra-cardiac information including malignancy, lung involvement or muscular burden | - Radiation exposure - Use as a monitoring tool is tricky due to radiation exposure - Requires dedicated fasting protocol for 12-18 hours prior to the scan - Requires expertise to perform and interpret |
| **Invasive modalities** | **EMB** | - Current gold standard - Can give a definitive diagnosis - Important to rule out specific forms of myocarditis (giant cell, eosinophilic) | - Invasive - Potential risk of complications (2-10% risk of major complication) - Should be performed at an experienced centre |

NT-proBNP, N-terminal pro-brain natriuretic peptide; ECG; electrocardiography; LV, left ventricle; CMR, cardiac magnetic resonance; LGE, late gadolinium enhancement; PET/CT, positron emission tomogaraphy/computed tomography; EMB endomyocardial biopsy
